# Nanoparticle-Mediated Genetic Transformation in a *Selaginella* Species

**DOI:** 10.3390/genes15081091

**Published:** 2024-08-19

**Authors:** Madhavi A. Ariyarathne, Beate Wone, Nisitha Wijewantha, Bernard W. M. Wone

**Affiliations:** 1 Department of Biology, University of South Dakota, Vermillion, SD 57069, USA; 2 Department of Physiology and Cell Biology, University of Arkansas for Medical Sciences, Little Rock, AR 72205, USA; 3 Department of Chemistry, University of South Dakota, Vermillion, SD 57069, USA

**Keywords:** *Selaginella*, genetic transformation, nanohydroxyapatites, desiccation tolerant

## Abstract

The genus *Selaginella* holds a key phylogenetic position as a sister species to vascular plants, encompassing desiccation-tolerant members. Some *Selaginella* species thrive in extremely arid conditions, enduring significant water loss and recovering upon rehydration. Consequently, *Selaginella* has emerged as a model system for studying desiccation tolerance in plant science. However, the absence of an efficient genetic transformation system has limited the utility of *Selaginella* species as a model. To address this constraint, we developed a nanoparticle-mediated transformation tool utilizing arginine-functionalized nanohydroxyapatites. This biocompatible system enabled the transient expression of the *GFP*, *GUS*, and *eYGFPuv* reporter genes in *Selaginella moellendorffii*. Establishing a stable genetic transformation technique for *S. moellendorffii* holds promise for application to other *Selaginella* species. This tool could be instrumental in identifying genetic resources for crop improvement and understanding genome-level regulatory mechanisms governing desiccation tolerance in *Selaginella* species. Furthermore, this tool might aid in identifying key regulatory genes associated with desiccation tolerance, offering potential applications in enhancing drought-sensitive crops and ensuring sustainable food production.

## 1. Introduction

The genus *Selaginella*, classified within the spike moss family Selaginellaceae of the lycophyte clade, occupies a pivotal phylogenetic position as a sister lineage to all vascular plants [1]. This genus includes approximately 700 species that thrive in a variety of ecosystems, ranging from tropical to arctic, temperate, and desert environments [1]. These species display a wide spectrum of desiccation tolerances [2]. Notably, at least 15 species within the genus, such as *S. lepidophylla* [3,4,5], *S. tamariscina* [6], *S. sellowii* [7], and *S. bryopteris* [8], are recognized for their desiccation tolerance capability. These species are particularly suited for studying the genetic mechanisms underlying plant vegetative desiccation tolerance, given their adaptive responses to desiccation and their seminal role in the evolution of the plant vascular system [1,4,9].

As *Selaginella* gains prominence as a model for elucidating desiccation tolerance and the evolution and function of plant vascular systems, the development of an effective genetic transformation methodology is imperative for unraveling the complex genetics of these traits. Currently, a genetic transformation protocol for *Selaginella* or any lycophytes is notably absent, which constrains their potential as model organisms. Addressing this gap, we formulated a nanoparticle-mediated genetic transformation protocol for the desiccation-sensitive *Selaginella moellendorffii*, using arginine-functionalized nanohydroxyapatite particles. This breakthrough suggests broader applicability, potentially extending to other *Selaginella* species, including those exhibiting desiccation tolerance like *S. lepidophylla*.

Plant genetic material delivery is challenged by the presence of a semi-rigid, multilayered cell wall. Traditional methods, such as *Agrobacterium*-mediated transformation, biolistic delivery, electroporation, and polyethylene glycol (PEG)-mediated delivery, each have their limitations [10]. *Agrobacterium*, though widely utilized due to its efficiency, is limited by species specificity and the randomness of DNA integration [11]. Biolistic delivery can target a broader range of species but risks tissue damage and random gene insertion [12]. Electroporation, despite being quick and cost-efficient, is only effective for a limited number of species and can be detrimental to cells [13,14]. PEG-mediated delivery, while efficient for protoplast transformation, often fails to regenerate protoplasts into whole, fertile plants, rendering it impractical for *in planta* transformations [13].

Nanoparticles present an ideal solution for delivering biomolecules across the plant cell wall without necessitating external forces [10], thus facilitating genetic modification without integrating transgenes [15,16]. Nanohydroxyapatite (nHA) particles, noted for their biodegradability, biocompatibility, non-toxicity, DNA protection from nuclease degradation, ease of synthesis, and modifiable surfaces for enhanced delivery [17], have emerged as promising nanocarriers for gene delivery in plants. These nanoparticles are efficiently metabolized by plants, providing nutritional benefits [18,19,20].

Transient transformation, often sufficient for gene function analysis, eliminates the need for stable transformants [21]. This method allows for the temporary introduction and expression of genes, enabling rapid and efficient studies without genome integration. Large-scale transient gene expression assays can be performed quickly, providing an effective alternative to the lengthy processes of stable transformation. Our study employs arginine-functionalized nanohydroxyapatite rods (R-nHAs) as biocompatible nanocarriers for the delivery of reporter genes into *S. moellendorffii* cells, demonstrating a non-damaging, non-toxic approach to transient plant genetic transformation.

## 2. Materials and Methods

### 2.1. Synthesis of Arginine-Functionalized Nanohydroxyapatite Particles

Nanohydroxyapatite (nHA) rods were synthesized following the protocol outlined by Izuegbunam et al. [17]. Initially, an aqueous solution of polyethylene glycol (PEG) at 1.5% (*w*/*w*) concentration was prepared. This PEG solution was subsequently combined with CaCl_2_ to obtain a 0.05 M solution with a volume of 100 mL. The mixture was allowed to equilibrate overnight at room temperature (RT) before being added dropwise to a 0.03 M aqueous solution of Na_2_HPO_4_, 100 mL in volume, under constant mechanical stirring at a speed of 1000 rpm. Following this step, the resultant solution was transferred to a sealed glass vial and incubated for 48 h at RT. Post-incubation, the solution underwent centrifugation at 11,000 rpm for 10 min to isolate the white precipitate formed. This precipitate was subjected to three successive washes with deionized water and another three with absolute ethanol, before being dried overnight at 60 °C. The dry powder was then calcined at 500 °C for 2 h, yielding the final nHA product.

Nanohydroxyapatite (nHA) rods (100 mg) underwent functionalization with arginine by being combined with a 0.1 wt% arginine solution (volume: 100 mL). The suspension was stirred at a speed of 600 rpm for 1 h to ensure thorough interaction. After stirring, the arginine-functionalized particles were separated *via* centrifugation at 8000 rpm for 10 min. They were then washed with deionized water to remove unbound arginine and centrifuged again under the same conditions. This washing step was repeated three times. Finally, the particles were collected by centrifugation and dried at room temperature overnight, yielding arginine-functionalized nanohydroxyapatite (R-nHA).

### 2.2. Plant Growth

*Selaginella moellendorffii* plants were purchased from Plants Delight Nursery Inc. (http://www.plantdelights.com) and were grown in Miracle-Gro^®^ Moisture Control^®^ Potting Mix (The Scotts Company LLC, Marysville, OH, USA) under a 16 h day/8 h night photoperiod at 22 °C in an environmentally controlled room.

### 2.3. The Cloning and Plasmid Isolation of the G3GFP-GUS Fusion Construct

The β-glucuronidase (GUS) gene, flanked by Att sites necessary for LR cloning, was synthesized by Gene Universal (Gene Universal Inc., Newark, DE, USA). An LR cloning reaction, employing the Gateway™ LR Clonase™ II Enzyme Mix (Invitrogen, Carlsbad, CA, USA), was conducted with the gateway binary vector pGWB452 (35S-G3GFP-R1-R2-Tnos) [22] and the GUS gene to create a G3GFP-GUS fusion construct (pGWB452-G3GFP::GUS). This construct allows for the expression of either reporter in transformed plants. The resulting pGWB452-G3GFP::GUS construct was then transformed into NEB^®^ 10-beta competent *E. coli* cells (strain C3019H) for amplification and/or storage (New England BioLabs, Ipswich, MA). Plasmid DNA was extracted from the *E. coli* cells using the Qiagen Plasmid Mini Kit (Qiagen, Hilden, Germany). Hereafter, the 11,833 bp pGWB452-G3GFP::GUS vector is referred to as pDNA.

### 2.4. Preparation of pGWB452-G3GFP::GUS|R-nHA Conjugate Solution

The procedure outlined by Izuegbunam et al. [17] was adapted to prepare any pDNA|R-nHA conjugates used in this study. Briefly, an aqueous suspension of R-nHA (1 mg/mL) was sonicated in an ice bath for 10 min to improve dispersion. A conjugate mixture was prepared using a 1:200 weight-to-weight (*w*/*w*) ratio of pDNA (3 μg) to R-nHA (600 μg). This mixture was agitated by flicking and inverting for 30 s without vortexing and subsequently incubated at 37 °C with shaking at 200 rpm for 90 min, with thorough mixing (thorough mixing was conducted by inverting the tubes several times and shaking by hand) every 30 min. A 10 mL aliquot of 0.5% low-viscosity carboxymethylcellulose (CMC) was introduced and stirred for 15 min at room temperature to maintain the pDNA|R-nHA conjugates in suspension, as per Liu and Lal [23]. The formation of the conjugates was confirmed by visualization on a 1% agarose gel.

### 2.5. Transient Transformation of pGWB452-G3GFP::GUS|R-nHA Conjugates in S. moellendorffii

Healthy, intact *S. moellendorffii* plantlets were incubated in small beakers containing pDNA|R-nHA conjugates dissolved in a 0.5% carboxymethyl cellulose (CMC) solution, with control groups incubated in a plain 0.5% CMC solution and in water only. The solutions underwent vacuum infiltration at −0.01 MPa for 1 min, following the procedure described by Izuegbunam et al. (2021) [17]. This process was repeated twice before the beakers with the infiltrated plantlets were transferred to a controlled environmental room. The infiltrated *S. moellendorffii* plantlets were maintained in the pDNA|R-nHA solution for 3 d prior to the assay for GFP (green fluorescent protein) or GUS (β-glucuronidase) expression.

### 2.6. Determination of GFP or GUS Reporter Gene Expression

Although the pGWB452-G3GFP::GUS construct permits the expression of either reporter in transformed plants, the same plant material cannot be used to visualize both reporters in the same cells. This limitation arises because the processes required to visualize GFP or GUS render the plant material too fragile for subsequent use. Consequently, different plant tissues must be used to visualize each reporter. For the analysis of GFP expression, sporophyll slides of *S. moellendorffii* were prepared, and GFP activity was visualized using a fluorescence microscope equipped with a Leica DFC3000 G camera (Leica Microsystems Inc., Buffalo Grove, IL, USA). A histochemical assay for GUS activity was conducted in line with the protocol of Lim et al. [24], with slight alterations. In brief, *S. moellendorffii* plantlets underwent vacuum infiltration at −0.07 MPa for 10 min using a GUS staining solution composed of 0.5 mg/mL X-Gluc (5-bromo-4-chloro-3-indolyl glucuronide) in 1 mL dimethylformamide, 50 mM sodium phosphate buffer at pH 7.0, 0.1 mM K_4_Fe(CN)_6_, 0.1 mM K_3_Fe(CN)_6_, and 4 mM EDTA, followed by an incubation at 37 °C overnight [24]. To improve the visibility of the GUS staining, treated samples were subsequently decolorized with 70% ethanol to remove chlorophyll. The processed samples were then examined with a Leica DM500 Binocular Microscope and a Leica EZ24 HD Stereo Microscope (Leica Microsystems Inc., Buffalo Grove, IL, USA).

### 2.7. The Plasmid Isolation of the pAXY001 Expression Clone That Contains the eYGFPuv Gene

The pAXY001 plasmid, which harbors the *eYGFPuv* gene as described by Yuan et al. [25], was acquired from www.Addgene.org (Addgene plasmid #179834). Initially received in bacterial stab form, the pAXY001 plasmid was streaked onto an agar plate containing the appropriate antibiotic to isolate single colonies. Subsequently, plasmid DNA was extracted from an individual colony using the Qiagen Plasmid Mini Kit (Qiagen, Hilden, Germany). The 14,360 bp vector will henceforth be designated as pDNA.

### 2.8. Preparation of pAXY001|R-nHA Conjugate Solution and Transient Transformation of pAXY001|R-nHA Conjugates into S. moellendorffii

The pDNA|R-nHA conjugate solution was prepared as per the method outlined previously, with a minor modification. Instead of coating with CMC, 10 mL of 0.1% *w*/*v* trimethyl chitosan (TMC) was utilized to coat the pDNA|R-nHA conjugates.

For the transient transformation of *S. moellendorffii*, healthy, intact plantlets were used. The *eYGFPuv* gene transient transformation assay involved applying 10 mL of the conjugate solution, containing 0.02% Silwet, with a 15 mL spray atomizer as per the protocols reported by Hu et al. and Thagun et al. [26,27]. The solution was sprayed uniformly until the plantlets were thoroughly covered. Subsequently, the treated *S. moellendorffii* plantlets were placed in small beakers with the pDNA|R-nHA conjugate solution and maintained in a controlled environment room. The plantlets remained in the solution for 3 d before the assessment of *eYGFPuv* gene expression. Control plantlets were similarly treated using a solution of R-nHAs that were coated in 0.1% TMC and incubated for 3 d. The expression of the *eYGFPuv* gene was visually detected under UV light using a uvBeast UVB-V3-365 (365 nm) according to Yuan et al. [25].

### 2.9. Agrobacterium-Mediated Delivery of GUS Reporter Gene

The β-glucuronidase (*GUS*) gene, complete with flanking attB sites needed for LR recombination, was synthesized by Gene Universal (Gene Universal Inc., Newark, DE, USA). This *GUS* gene was subsequently cloned into the Gateway^®^ binary vector [22] using the Gateway™ LR Clonase™ II Enzyme Mix (Invitrogen, Carlsbad, CA, USA), culminating in the construction of pGWB402-GUS. The resultant recombinant plasmid was introduced into 10-beta competent *Escherichia coli* cells (New England Biolabs, Ipswich, MA, USA) via heat-shock transformation. Plasmids were then isolated from the *E. coli* cells using the Qiagen Plasmid Mini Kit (Qiagen, Hilden, Germany).

The pGWB402-GUS vector was further transformed into *Agrobacterium tumefaciens* strain GV3101 by the freeze–thaw method [28]. A preparatory step involved cultivating a single *Agrobacterium* colony in liquid Luria–Bertani (LB) medium with the appropriate antibiotics, 24 h before the transformation. This precursor culture was incubated at 28 °C with shaking at 250 rpm for 2 d. After incubation, the culture was centrifuged at 3000 rpm for 10 min at room temperature, and the supernatant was discarded. The resultant pellet was resuspended in infiltration buffer (10 mM MES, 10 mM MgCl_2_, pH 5.6). The centrifugation and resuspension cycles were performed thrice to eliminate any residual LB medium, thereby arresting *Agrobacterium* growth. Finally, the bacterial pellet was resuspended in infiltration buffer to achieve a 1:10 dilution (OD600 = 0.5), and acetosyringone was added to a final concentration of 200 µM to facilitate infiltration.

Healthy, intact *S. moellendorffii* plantlets were placed in small beakers with the infiltrate. The solution was subjected to vacuum infiltration at −0.01 MPa for 1 min, in accordance with the protocol established by Izuegbunam et al. (2021), and this step was replicated twice before the plantlets were relocated to a controlled environment chamber [17]. The *S. moellendorffii* plantlets were incubated in the infiltrate for 3 d before the assessment of GUS gene expression was conducted.

### 2.10. Characterization of Conjugates

The conjugates composed of plasmid DNA and arginine-functionalized nanohydroxyapatite (pDNA|R-nHA—refer above for their preparation), both in aqueous suspension and those coated with carboxymethyl cellulose (CMC) (pDNA|R-nHA-CMC) and trimethyl chitosan (TMC) (pDNA|R-nHA-TMC), were lyophilized for 24 h under conditions of 0.05 mbar and −89 °C. This freeze-drying process was essential for subsequent characterization via transmission electron microscopy (TEM) and zeta potential analysis. TEM was employed to assess alterations in the morphology, size, shape, and the degree of agglomeration of the prepared conjugates. Zeta potential measurements were conducted to verify the binding of CMC and TMC to the pDNA|R-nHA conjugates.

#### 2.10.1. Transmission Electron Microscopy Imaging

Conjugate morphologies were examined using an FEI Tecnai G2 TWIN Transmission Electron Microscope (TEM) operating at an accelerating voltage of 200 kV (Field Electron and Ion Company, Hillsboro, OR, USA). A monodispersed conjugate solution (1 mg/mL) was diluted 20-fold with Nanopure water. Subsequently, a drop of this diluted solution was placed onto copper-coated TEM grids, which were then left to air-dry for 2 d within a vacuum desiccator.

#### 2.10.2. Zeta Potential Measurements

Zeta potential measurements were performed using a Malvern Zetasizer Nano ZS (Malvern Panalytical Ltd., Malvern, UK). For these measurements, conjugates (1 mg) were dispersed in 10 mL of phosphate-buffered saline (PBS, pH 7.4, 0.1 M) through 10 min of sonication with an ultrasonication probe. The resulting suspension (0.1 mg/mL in PBS, pH 7.4) was then transferred to a disposable capillary zeta cell for zeta potential analysis. Each sample underwent five individual zeta potential determinations, with each determination comprising ten runs. The reported zeta potential values represent the mean of these determinations. The conductivity of the PBS suspension consistently exceeded 0.8 mS/cm.

## 3. Results

### 3.1. Arginine-Functionalized Nanohydroxyapatite Particle-Mediated Expression of GFP or GUS Reporter Genes in S. moellendorffii Sporophylls

**Figure 1:**
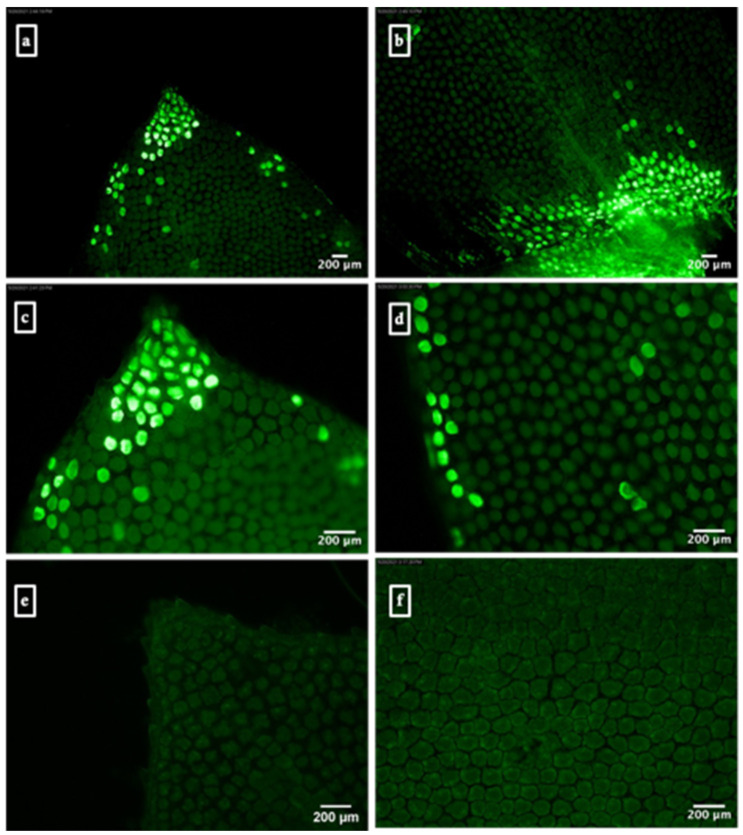
Arginine-functionalized nanohydroxyapatite (R-nHA)-mediated transient expression of GFP in *Selaginella moellendorffii* sporophylls. (**a**–**d**) GFP fluorescence in *S. moellendorffii* sporophylls 3 d post-R-nHA-mediated *in planta* transformation, observed using a Leica DMRA2 fluorescence microscope coupled with a Leica DFC3000 G camera. (**e**) Treated control sporophylls 3 d post-incubation in a solution containing R-nHAs coated with 0.5% carboxymethylcellulose (CMC). (**f**) Untreated control sporophylls incubated in water. Magnifications: 100× for (**a**,**b**), 200× for (**c**–**f**) (n = 6).

**Figure 2:**
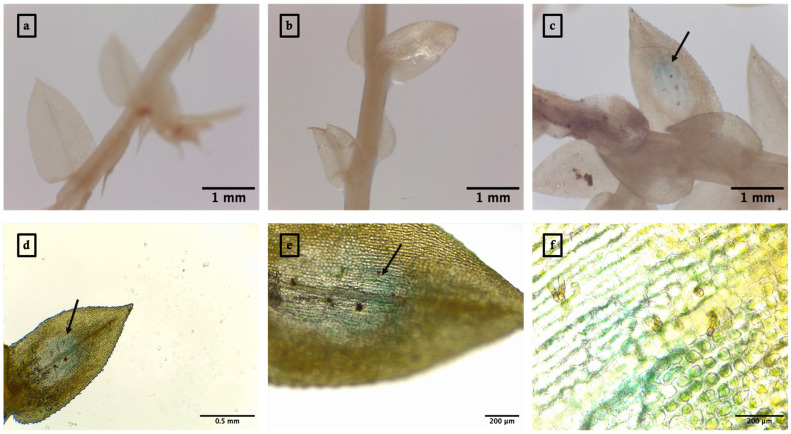
Arginine-functionalized nanohydroxyapatite (R-nHA)-mediated transient expression of GUS in *Selaginella moellendorffii* sporophylls. (**a**) Untreated control sporophyll 3 d post-incubation in water. (**b**) Control sporophyll 3 d post-incubation in a solution of R-nHAs coated with 0.5% low-viscosity carboxymethylcellulose. (**c**–**f**) Sporophylls showing transient GUS expression 3 d after R-nHA-mediated *in planta* transformation. Imaging was performed with a Leica EZ24 HD Stereo Microscope for (**a**–**c**) and a Leica DM500 Binocular Microscope for (**d**–**f**). Magnifications: 35× for (**a**–**c**), 40× for (**d**), 100× for (**e**), and 400× for (**f**). Blue spots or patches (arrows) represent the histochemical staining of GUS (n = 10).

Green fluorescent protein (GFP) expression was observed as green patches within the cytoplasm of *S. moellendorffii* sporophylls infiltrated with R-nHA-mediated pGWB452-G3GFP::GUS plasmid DNA. These observations were made using a Leica DMRA2 fluorescence microscope equipped with a Leica DFC3000 G camera (Leica Microsystems Inc., Buffalo Grove, IL, USA) (Figure 1). Similarly, the transient expression of the GUS gene was evidenced by the presence of blue spots or patches in the infiltrated plant tissues (Figure 2).

### 3.2. Arginine-Functionalized Nanohydroxyapatite Particle-Mediated Expression of eYGFPuv Reporter Gene in S. moellendorffii Sporophylls

**Figure 3:**
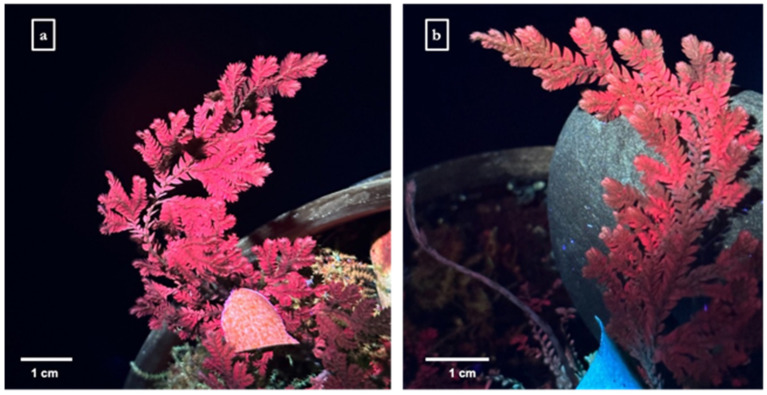
Arginine-functionalized nanohydroxyapatite (R-nHA)-mediated *in planta* transient expression of the green fluorescent protein (eYGFPuv) in *Selaginella moellendorffii* sporophylls. (**a**) Control *S. moellendorffii* sporophyll observed under UV light 3 d post-spraying of a solution containing R-nHA coated with 0.1% trimethyl chitosan. (**b**) Treated sporophylls exhibiting eYGFPuv expression under UV light 3 d post-R-nHA-mediated spraying of the *eYGFPuv* gene, appearing brownish due to the overlay of red autofluorescence and green fluorescence. In contrast, control sporophylls display distinctively red autofluorescence (n = 4).

The enhanced green fluorescent protein variant (eYGFPuv) was optimized for maximal fluorescence visibility under ultraviolet (UV) light without the need for specialized microscopy [25]. Three days post-infiltration with R-nHA-mediated pDNA containing the *eYGFPuv* gene, green fluorescence was observed under UV light on *S. moellendorffii* sporophylls. In contrast, untransformed tissues exhibited red autofluorescence (Figure 3a). Notably, the combination of eYGFPuv green fluorescence with the red autofluorescence of the tissues resulted in a brownish hue (Figure 3b), whereas the control *S. moellendorffii* sporophylls showed only distinct red autofluorescence under UV light.

### 3.3. Agrobacterium-Mediated Transient Expression of GUS in S. moellendorffii Sporophylls

**Figure 4:**
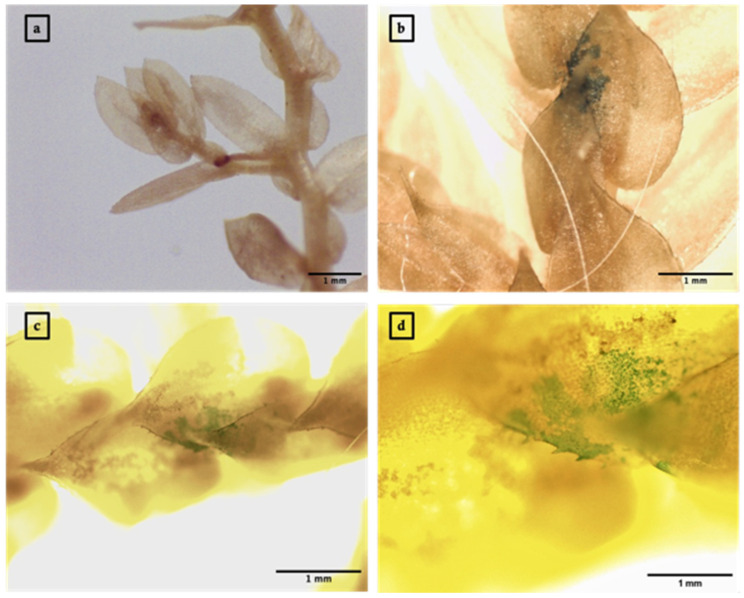
*Agrobacterium*-mediated transient expression of GUS in *Selaginella moellendorffii* sporophylls. (**a**,**b**) Sporophylls imaged with a Leica EZ24 HD Stereo Microscope at magnifications of 25× for (**a**) and 35× for (**b**). (**c**,**d**) Sporophylls imaged with a Leica DM500 Binocular Microscope at magnifications of 40× for (**c**) and 100× for (**d**). Blue spots or patches represent the histochemical staining of GUS (n = 8).

The transient expression of GUS in *S. moellendorffii* sporophylls was achieved through *Agrobacterium*-mediated transformation. Observations were made using a Leica EZ24 HD Stereo Microscope and a Leica DM500 Binocular Microscope. Three days post-infiltration, sporophylls underwent histochemical staining for GUS and subsequent chlorophyll clearing with ethanol. This resulted in the visualization of characteristic blue spots or patches within the sporophyll tissues (Figure 4).

### 3.4. Characterization of Conjugates

**Figure 5:**
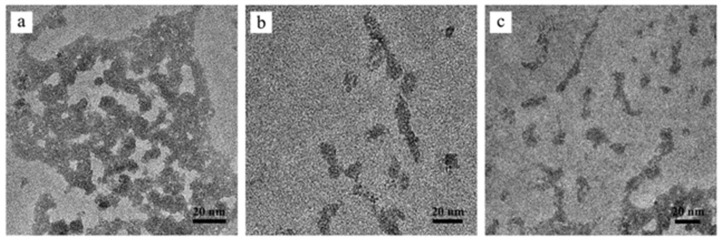
The morphological characterization of plasmid DNA|arginine-functionalized nanohydroxyapatite (pDNA|R-nHA) conjugates. Transmission electron microscopy (TEM) images showing the morphology of (**a**) pDNA|R-nHA conjugates, (**b**) pDNA|R-nHA conjugates coated with 0.5% low-viscosity carboxymethylcellulose (CMC), and (**c**) pDNA|R-nHA conjugates coated with 0.1% trimethyl chitosan (TMC). Observations were made using an FEI Tecnai G2 TWIN TEM operated at an accelerating voltage of 200 kV.

**Figure 6:**
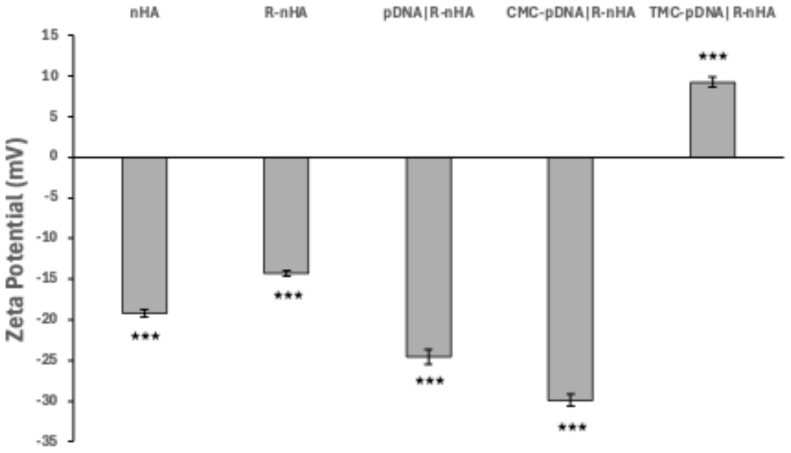
Zeta potential measurements of nanohydroxyapatites (nHAs). Zeta potential values of unfunctionalized nHA, arginine-functionalized nHAs (R-nHAs), and plasmid DNA (pDNA)|R-nHA conjugates with and without polymer coatings of 0.5% low-viscosity carboxymethylcellulose (CMC) or 0.1% trimethyl chitosan (TMC) (CMC-pDNA)|R-nHA and TMC-pDNA)|R-nHA) at pH 7.4 (n = 5, values represent means ± SD, *** adjusted *p* < 0.001, one-way ANOVA with Tukey’s HSD multiple comparisons).

Transmission electron microscopy (TEM) imaging revealed a notable reduction in particle agglomeration when the conjugates were coated with carboxymethylcellulose (CMC) or trimethyl chitosan (TMC), suggesting an effective mitigation of particle aggregation by these stabilizing agents (Figure 5). Zeta potential measurements indicated significant surface charge alterations of the conjugates (Figure 6). The initial nanohydroxyapatite (nHA) nanoparticles displayed a slight negative charge (−19.20 mV). Functionalization with arginine decreased this charge, resulting in a zeta potential of −14.33 mV for R-nHA nanoparticles. The binding of pDNA to R-nHA nanoparticles further increased the negative charge, as evidenced by a zeta potential of −24.53 mV for pDNA-R-nHA conjugates. Coating with CMC augmented this negative charge, yielding a zeta potential of −29.89 mV. In contrast, TMC coating produced a positive zeta potential of 9.27 mV.

## 4. Discussion

The use of R-nHA rods in plant gene delivery has emerged as a promising and facile method for the introduction of exogenous genes into plant cells. Comprising calcium and phosphate ions, nHA particles mimic the calcium-to-phosphate ratio found in mammalian bones and teeth, making them highly biocompatible with living systems [29]. This technique offers several advantages, including cost-effectiveness, simplicity, rapidity, species independence, scalability, and minimal damage to target plants [17,20,21]. Like single-walled carbon nanotube-mediated gene delivery, nHA-mediated gene delivery avoids the undesirable integration of vector sequences into the target genome, thereby preserving the integrity of the native genetic material [15,17]. Additionally, nanohydroxyapatite nanocarriers are biodegradable and pose a reduced risk of horizontal gene transfer compared to other nanocarriers, such as single-walled carbon nanotubes. Crucially, our system employs a non-toxic approach that ensures plant viability while achieving effective gene delivery, aligning with the recently reported non-toxic, nanoparticle-based plant transformation method described by Ahmed et al. [30].

To enhance the biocompatibility and efficiency of nanoparticle-mediated gene delivery in plants, we utilized arginine-functionalized nHAs (R-nHAs). The cationic nature of arginine facilitates electrostatic interactions with negatively charged nucleic acids, thereby improving the binding and cellular uptake of plasmid-loaded nanoparticles [20,31]. Using arginine, a naturally occurring amino acid, mitigates potential toxicity concerns associated with nanoparticle functionalization.

Our zeta potential measurements indicated surface charge alterations in the conjugates. Initially, nHA rods exhibited a slight negative charge due to hydroxyl groups on their surface. Arginine functionalization altered the zeta potential of R-nHA rods to less negative values, confirming successful arginine attachment. The subsequent binding of plasmid DNA (pDNA) to R-nHA further increased the negative charge, indicating effective pDNA binding through electrostatic interactions.

We enhanced gene delivery by coating pDNA|R-nHA conjugates with stabilizing agents, namely carboxymethylcellulose (CMC) and trimethyl chitosan (TMC). CMC-coated conjugates exhibited a highly negative zeta potential, confirming effective CMC incorporation, while TMC coating resulted in a positive zeta potential, indicative of TMC integration. The contrasting charges of CMC and TMC contributed to improved electrostatic stabilization, enhancing particle dispersibility, stability, and, consequently, gene delivery efficiency [32,33].

The use of CMC and TMC coatings significantly reduced particle aggregation in the R-nHA-mediated gene delivery system, thereby improving its effectiveness. Uncoated conjugates exhibited aggregation, likely due to electrostatic interactions and van der Waals forces. However, CMC and TMC coatings effectively mitigated particle aggregation, enhancing the efficacy of gene delivery. Notably, TMC, a biocompatible and biodegradable polysaccharide derivative, has been recognized for its utility in gene delivery [33]. The coating process maintained the size and shape of the pDNA|R-nHA conjugates, which is crucial for efficient cellular uptake and gene delivery in plants [17]. The resulting coated conjugates demonstrated stable, well-dispersed characteristics, likely improving their interaction with plant cell walls and membranes, thereby enhancing transformation efficiency. In summary, the coated R-nHA-mediated gene delivery system successfully facilitated the transient transformation of the *GFP*, *GUS*, and *eYGFPuv* reporter genes into *S. moellendorffii*.

The current study reports the development of a simple, fast, and reproducible method for transiently introducing genes of interest into *S. moellendorffii* using R-nHA rods. However, despite successfully expressing reporter genes in *S. moellendorffii* sporophylls, we encountered challenges with the *in planta* visualization of *eYGFPuv* expression using the spray method. This difficulty arose from the inherent autofluorescence of *S. moellendorffii* sporophylls under UV exposure, which diluted the bright green fluorescence indicative of eYGFPuv protein expression. The overlap of green fluorescence with red autofluorescence produced a brownish hue, complicating the clear assessment of *eYGFPuv* gene expression. This finding suggests limitations in the efficacy of the nano-enabled transformation *via* the spray method, particularly when compared to the efficiency of *Agrobacterium*-mediated transformation as reported in a study by Thagun et al. [27]. Consequently, our overall findings suggest that the nano-enabled transformation method might not be as effective as *Agrobacterium*-mediated transformation in general due to inherent differences in the delivery and expression mechanisms of exogenous genes. Unlike *Agrobacterium*-mediated transformation, plasmids delivered by nanoparticles lack virulence factors and replicative ability. This disparity appears to reduce target gene expression levels and clarity in visualizing the expressed eYGFPuv protein, as observed in our study. These limitations highlight the need for further refinements of nano-mediated gene delivery techniques.

*Selaginella* species, owing to their evolutionary significance, innate desiccation tolerance, and experimental tractability, have emerged as ideal model organisms for investigating desiccation tolerance and plant abiotic stress survival strategies [21]. Diverging from the principal lineage of flowering plants over 400 million years ago [1], these species exhibit distinct characteristics and survival mechanisms that have been conserved throughout their evolutionary history [34]. Research on *Selaginella* species not only sheds light on the early evolution of land plants but also elucidates the adaptive strategies that facilitated their terrestrial colonization. Moreover, attributes such as a compact genome, short life cycle, and ease of laboratory cultivation render *Selaginella* species practical and efficient models for exploring desiccation tolerance and other biological aspects [21,35]. In this context, the nano-biomimetic carrier system we developed for *S. moellendorffii* opens new avenues for stable genetic transformation.

## 5. Conclusions

The reported transformation system has the potential to revolutionize genetic studies in *Selaginella* species. Dipping or spraying germline tissues (i.e., strobili) with a genome-editing plasmid/nHA conjugate solution could facilitate the generation of T_0_ gametophytes with stable genetic alterations. The ability to achieve stable transformation in *Selaginella* species might pave the way for detailed molecular investigations into desiccation tolerance mechanisms. By employing gene knockout strategies, researchers can dissect the roles of specific genes in this trait, unraveling the intricate pathways involved. Such insights are vital for a comprehensive understanding of desiccation tolerance processes. Furthermore, this knowledge has the potential to inform strategies aimed at enhancing drought resistance in a broader range of plant species, offering significant implications for agricultural sustainability.

## Data Availability

No datasets were generated or analyzed during the current study.
